# Psychometric properties of quality of life questionnaires for patients with breast cancer-related lymphedema: A protocol for a systematic review

**DOI:** 10.1097/MD.0000000000023897

**Published:** 2020-12-24

**Authors:** Estu Meilani, Asfarina Zanudin, Nor Azlin Mohd Nordin

**Affiliations:** Physiotherapy Programme, Centre for Rehabilitation and Special Needs Studies, Faculty of Health Sciences, Universiti Kebangsaan Malaysia, Malaysia.

**Keywords:** breast cancer-related lymphedema, psychometric properties, quality of life, questionnaire

## Abstract

**Background:**

Breast-cancer related lymphedema (BCRL) is a common condition among breast cancer survivors that could impact the quality of life (QoL) of patients. Exploring the QoL of the patients with BCRL using valid and reliable QoL is crucial to capture the status of this important aspect hence appropriate intervention could be implement to patient. However, so far no scientific review is available, which reports the psychometric properties of the QoL questionnaires used in BCRL. The purpose of this systematic review is to comprehensively assess the psychometric properties of QoL questionnaires in patients with BCRL.

**Methods:**

We will perform comprehensive searches of published studies in electronic databases such as Medline (via Ovid), EBSCOhost, PubMed, Scopus, and Web of Science by using the following search terms: “quality of life”; “breast cancer”; “upper limb”; “lymphedema”; “questionnaire”; and “measurement properties.” Only full-text articles in English language are included. Two reviewers will independently conduct the article selection, data extraction, and quality assessment. Any possible conflict between the 2 reviewers is going to be solved with the help of a third reviewer. The Consensus-based Standards for the Selection of Health Measurement Instrument (COSMIN) checklist and manual will be used to assess the selected study quality.

**Results:**

This review will provide an updated overview of available lymphedema-specific questionnaires used in BCRL population and then recommend the most valid and reliable QoL questionnaire for clinical and research use in patients with BCRL.

**Conclusion:**

This review may help the clinician and researcher to find an updated overview of various questionnaires used to assess BCRL patients’ QoL.

**Ethics and dissemination:**

This review will use data from published studies. Therefore, ethical approval is not required prior to this review. The results of this review will be published in a peer-reviewed journal or presented at conferences.

**Study Registration:**

OSF osf.io/8xwym.

## 1 Introduction

Breast cancer is the most common cancer diagnosis that impacts over 2,000,000 women and causes the most deaths among women worldwide.^[1]^ Despite improvements in many treatment options for breast cancer, lymphedema remains a significant comorbidity of post-breast cancer treatments.^[2,3]^ Lymphedema is a chronic pathological condition resulting from an over-accumulation of protein-rich fluid in extracellular space due to low output failure of the lymphatic system. Breast cancer-related lymphedema (BCRL) mostly occur after 2 or 3years following the treatment procedures, with the overall incident rate ranging from 8% to 56%. There are a few factors that can increase the risk of breast cancer survivors developing lymphedema, such as emerging scar from the surgical intervention,^[4]^ having more lymph nodes removed,^[5,6]^ receiving chemotherapy,^[7]^ receiving radiotherapy,^[8–10]^ being obese, and being married.^[7]^

BCRL patients often reported a feeling of heaviness and tightness, discomfort, pain, or stiffness in the affected arm. The increased size of the affected arm in patients with BCRL might lead to further impairments in physical, psychological, and social well-being.^[4,11]^ Complications in these 3 aspects would then decrease the patients’ ability to work efficiently and reduce their quality of life (QoL).^[12,13]^ Hence, it is particularly important to measure, evaluate, and if possible improve the QoL of patients suffering from BCRL.^[14–17]^ While QoL has been assessed in patients with BCRL, it is so far unknown as to which questionnaire has the best psychometric properties. Psychometric properties allude the validity and reliability of the measurement tool. Validity and reliability are the 2 prerequisites of measurement which are equally important as without them, health practitioners will not be able to confidently draw accurate conclusions from the collected data. Comprehensive assessment is required in order to establish the validity and reliability of a questionnaire.^[18]^

Consensus-based standards for the selection of health measurement instruments (COSMIN) steering committee developed a comprehensive methodological guideline for systematic reviews of patient-reported outcome measures (PROMs).^[19]^ This guideline was established in accordance with the existing guidelines for reviews, such as the Cochrane Handbook for systematic reviews of intervention ^[20]^ and for diagnostic test accuracy reviews,^[21]^ the PRISMA statement,^[22]^ the Institute of Medicine (IOM) standards for systematic reviews of comparative effectiveness research,^[23]^ and the Grading of Recommendations Assessment, Development and Evaluation (GRADE) principles.^[24]^ The COSMIN risk of bias checklist which we will utilize in this review is one of 3 versions of the original COSMIN checklists. This checklist is a proprietary developed for assessing the methodological quality of a study by providing standards referring to design requirements and preferred statistical methods of each measurement properties.^[25]^

A few systematic reviews in studies assessing the QoL questionnaires have been conducted.^[13,26,27]^ Nevertheless, these studies were either: not focused on studies which only assess psychometric properties^[13]^; do not include an adequate number of questionnaire, but focused only on 1 generic questionnaire (SF-36) instead ^[26]^; not focused on the BCRL population^[13,26]^; or not comprehensively assessing the psychometric properties using a specific checklist.^[27]^ Thus, our systematic review aims to assess the psychometric properties quality of various questionnaires assessing QoL in BCRL patients by using an exclusively designed COSMIN checklist.^[19]^ Ultimately, based on this review, we will recommend the most valid and reliable questionnaire for future uses in both research and clinical practice.

## 2 Methods

### 2.1 Study protocol registration

This study protocol has been registered in Open Science Framework/OSF (osf.io/8xwym). The protocol was drafted based on the guidelines outlined in the Preferred Reporting Items for Systematic Reviews and Meta-Analysis Protocol (PRISMA-P).^[20]^

### 2.2 Study inclusion and exclusion criteria

Only full-text articles in English language which assessed psychometric properties of the QoL questionnaires in BCRL patients are included in this review. Studies that use either original or translated version of patient-reported questionnaires are also included. The systematic review would focus on the taxonomy developed by COSMIN which covers 3 main domains and related measurement properties, which are reliability (internal consistency and measurement error), validity (content validity, construct validity, and criterion validity), and responsiveness. Identified studies which assess 1 or more of these measurement properties are included.

However, studies which only consist of abstract, dissertation, conference proceedings, editorials, opinion pieces, review papers, letters, single case studies, short communications, and technical notes are excluded. Furthermore, studies in healthy population and/or studies which its main purpose is not to assess psychometric properties as defined above are also excluded from the systematic review.

### 2.3 Article sources and search strategy

Articles search will be conducted using electronic databases such as Ovid MEDLINE, EBSCOHost, PubMed, Scopus, and Web of Sciences by using the following search terms: “quality of life”, “breast cancer or carcinoma”, “upper limb or extremity”, “lymphedema”, “questionnaire”, and “measurement properties”. An exclusion filter for measurement properties developed by Terwee et al ^[28]^ will also be used in the search process. More detailed search strategy are provided in Table 1. Articles will be searched and retrieved from the mentioned databases from the inception until present. The reference list of identified records will then be screened for duplicates.

**Table 1:** Search strategy for OVID medline.

No	Search terms
#1	(quality of life OR quality of living OR life quality OR welfare OR standard of living OR well-being)
#2	(breast cancer OR mammary cancer OR breast carcinoma)
#3	(upper limb OR upper extremity OR arm OR hand)
#4	(lymphedema OR lymphoedema OR lymphodema)
#5	(questionnaire OR survey or inquiry OR question sheet OR enquiry)
#6 (developed by Terwee et al^[28]^)	(measurement properties) OR (accuracy) OR (accurate) OR (clinimetr^∗^) OR (coefficient^∗^) OR (consisten^∗^) OR (correlated) OR (correlation^∗^) OR (cronbach) OR (discrimina^∗^) OR (interrater) OR (inter-rater) OR (intersession) OR (inter-session) OR (intertester) OR (inter-tester) OR (Intrarater) OR (intra-rater) OR (intratester) OR (intra-tester) OR (kappa) OR (Observer variation) OR (predictiv^∗^) OR (propert^∗^) OR (Psychometrics) OR (psychometr^∗^) OR (reliab^∗^) OR (repeatable) OR (repeatability) OR (Reproducibility of Results) OR (reproducible) OR (reproducibility) OR (responsive^∗^) OR (Sensitivity and Specificity) OR (sensitive) OR (sensitivity) OR (spearman^∗^) OR (specific) OR (specificity) OR (spearman) OR (subscale^∗^) OR (suitable) OR (suitability) OR (test development) OR (test-retest) OR (useful^∗^) OR (utility) OR (valid) OR (validity) OR (validat^∗^) OR (Validation studies)
Combination search	#1 AND #2 AND #4 AND #5 AND #6 AND NOT #7
Limiters	HUMANS, ENGLISH, JOURNAL, ARTICLE

### 2.4 Study selection process

**Figure 1:**
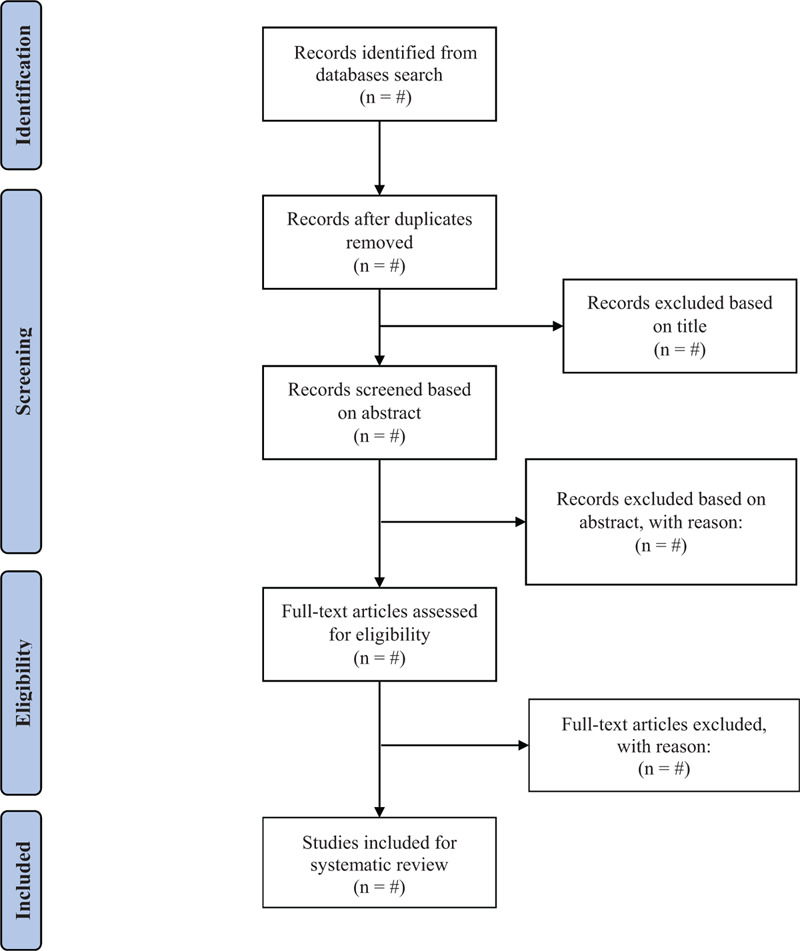
PRISMA flow charts of the study selection process (notes: “#” = unspecified number).

After removing the duplicates, the list of identified articles will first be reviewed for its title followed by its abstract. Full-text articles will then be retrieved and examined to obtain a final list of eligible studies. The study selection will be done in concordance with the PRISMA flow chart for systematic review and meta-analysis (see Fig. 1).

### 2.5 Data extraction

A data extraction sheet containing information about the characteristics of each questionnaire in the included studies will be developed. The characteristics that will be extracted from the included questionnaires comprise of the name of the questionnaires, the references to the article from which the questionnaire was developed, the constructs which were measured, the language used and the study of population for which the questionnaires were targeted, the number of scale or subscales and the number of items included, the response options used, and the information on recall period. The extracted characteristics of the included sample(s) will consist of geographical location, language, important disease characteristics, target population, sample size, age, gender, and setting. This information is needed to determine whether the results of different studies are sufficiently similar to be summarized.

### 2.6 Quality assessment

The quality of all eligible full-text articles, which represent the studies will be assessed by using the COSMIN checklist and scoring manual. The methodological quality of each study is assessed by using the corresponding boxes in COSMIN Risk of Bias (RoB) checklist. It contains 10 boxes with standards for PROM development (box 1) and for 9 measurement properties which are content validity (box 2), structural validity (box 3), internal consistency (box 4), cross-cultural validity/measurement invariance (box 5), reliability (box 6), measurement error (box 7), criterion validity (box 8), hypotheses testing for construct validity (box 9), and responsiveness (box 10). The number of items in each box varies from 4 to 35 items with a four-point rating system which are, “V = very good”, “A = adequate”, “D = doubtful”, and “I = inadequate”.^[25]^

Subsequently, the result of each study on a measurement property will be rated against the updated criteria for good measurement properties. After assessing a single study, the next step is to evaluate the questionnaire as a whole. The summarized results per measurement properties per questionnaire would again be rated against the same quality criteria for good measurement properties (see Table 2). The next step is to grade the quality of evidence, which refers to the confidence that the pooled or summarized result is trustworthy. The grading of the quality is based on the Grading of Recommendation Assessment, Development, and Evaluation (GRADE) approach for systematic reviews of clinical trials (see Table 3).^[24]^

**Table 2:** Criteria for good measurement properties^[19]^.

Measurement properties	Rating	Criteria
Structural validity	+	CTT:CFA: CFI or TLI or comparable measure >0.95 OR RMSEA <0.06 OR SRMR <0.08^2^IRT/Rasch:No violation of unidimensionality: CFI or TLI or comparable measure >0.95 OR RMSEA <0.06 OR SRMR <0.08^2^*AND*no violation of monotonicity: adequate looking graphs OR item scalability >0.30*AND*adequate model fit:IRT: χ^2^ > 0.01Rasch: infit and outfit mean squares ≥0.5 and ≤1.5 OR Z-standardized values >-2 and <2
	?	CTT: Not all information for ‘+’ reportedIRT/Rasch: Model fit not reported
	−	Criteria for ‘+’ not met
Internal consistency	+	At least low evidence for sufficient structural validity AND Cronbach's alpha(s) ≥0.70 for each unidimensional scale or subscale
	?	Criteria for “At least low evidence for sufficient structural validity” not met
	−	At least low evidence for sufficient structural validity AND Cronbach's alpha(s) < 0.70 for each unidimensional scale or subscale
Reliability	+	ICC or weighted Kappa ≥ 0.70
	?	ICC or weighted Kappa not reported
	−	ICC or weighted Kappa < 0.70
Measurement error	+	SDC or LoA < MIC
	?	MIC not defined
	−	SDC or LoA > MIC
Hypothesis testing for construct validity	+	The result is in accordance with the hypothesis
	?	No hypothesis defined (by the review team)
	−	The result is not in accordance with the hypothesis
Cross-cultural validity/measurement invariance	+	No important differences found between group factors (such as age, gender, language) in multiple group factor analysis OR no important DIF for group factors (McFadden's R^2^ < 0.02)
	?	No multiple group factor analysis OR DIF analysis performed
	−	Important differences between group factors was found
Criterion validity	+	Correlation with gold standard ≥ 0.70 OR AUC ≥ 0.70
	?	Not all information for ‘+’ reported
	−	Correlation with gold standard < 0.70 OR AUC < 0.70
Responsiveness	+	The result is in accordance with the hypothesis OR AUC ≥ 0.70
	?	No hypothesis defined (by the review team)
	−	The result is not in accordance with the hypothesis OR AUC < 0.70

**Table 3:** Definitions of quality levels of Modified GRADE approach for grading the quality of evidence ^[25]^.

Quality level	Definitions	Lower if
High	We are very confident that the true measurement property lies close to that of the estimate of the measurement properties	Risk of bias-1 Serious-2 Very serious-3 Extremely serious
Moderate	We are moderately confident in the measurement property estimate; the true measurement property is likely to be close to the estimate of the measurement property, but there is a possibility that it is substantially different	Inconsistency-1 Serious-2 Very serious
Low	Our confidence in the measurement property estimate is limited; the true measurement property may be substantially different from the estimate of the measurement property	Imprecision-1 total n = 50 – 100-2 total n < 50
Very low	We have very little confidence in the measurement property estimate; the true measurement property is likely to be substantially different from the estimate of the measurement property	Indirectness-1 Serious-2 Very serious

## 3 Discussion

Altough there are many treatment options for breast cancer, treatments such as mastectomy, radiotherapy, chemotherapy, and axillary node dissection often cause damage in the lymphatic system and is believed to be one of factors which result in the lymphedema development.^[2]^The risk of lymphedema among married women is 1.36 higher compared to unmarried women which might be related to the types of activities married women engage in (e.g., more routine household chores, care of children, etc.). ^[7]^

Assessment of quality of life is an important aspect in the treatment series of lymphedema since this disease can significantly impact QoL in the affected individuals. Patients with lymphedema experience various symptoms including swelling, pain, restricted joint mobility, skin thickness, pain,^[29]^ depression, anxiety, and negative body image.^[30]^ These physical and psychological symptoms substantially affect QoL, which then result in patients’ inability to stay active at work and home. A qualitative Swedish study using a phenomenological method, investigated breast cancer survivors with arm lymphedema found that these women had difficulties in coping with attitudes of their surroundings and also with the chronic disease itself. The difficulties in coping with their physical changes and inability to do things they used to do may cause stress to some of them.^[14]^ Another study using European Organization for Research and Treatment of Cancer Quality of Life Breast Cancer-Specific version (EORTC QLQ-BR23) reported that breast cancer survivors with arm lymphedema were more disabled, had lower QoL, and experienced more psychological distress compared to those breast cancer survivors without lymphedema^[17]^ and this result is in agreement with a previous study.^[15]^ A study by Jager et al assessed 80 breast cancer women with and without lymphedema by using Frankfurt Body Image Questionnaire and 36-Item Short-Form Health Survey (SF-36). The result indicated that women with lymphedema had lower scores for both body image and QoL.^[16]^ A majority of studies assessed in a systematic review by Pusic et al stated that patients with BCRL showed significantly poorer QoL outcomes, which included physical functioning, psychological, and social well-being.^[13]^

A recent systematic review by Cornelissen et al aimed to investigate the most complete and accurate questionnaires that assess QoL in patients with BCRL and the review recommended the Lymphedema Quality of Life Inventory (Ly-QLI) and Lymphedema Functioning Disability and Health Questionnaire (Lymph-ICF).^[27]^ However, to date, no review is available which reports the psychometric properties of QoL questionnaires for patients with BCRL. Therefore, to our knowledge, this is the first review to systematically assess the psychometric properties quality of QoL questionnaire in BCRL populations. Findings of this review can be used to implicate clinical practice and future studies targeting patients with BCRL.

## Author contributions

**Conceptualization:** Estu Meilani, Asfarina Zanudin, Nor Azlin Mohd Nordin.

**Data curation:** Estu Meilani.

**Formal analysis:** Estu Meilani.

**Funding acquisition:** Nor Azlin Mohd Nordin.

**Investigation:** Estu Meilani.

**Methodology:** Estu Meilani, Asfarina Zanudin, Nor Azlin Mohd Nordin.

**Project administration:** Asfarina Zanudin.

**Resources:** Estu Meilani.

**Software:** Estu Meilani.

**Supervision:** Asfarina Zanudin, Nor Azlin Mohd Nordin.

**Validation:** Asfarina Zanudin, Nor Azlin Mohd Nordin.

**Visualization:** Estu Meilani, Asfarina Zanudin, Nor Azlin Mohd Nordin.

**Writing – original draft:** Estu Meilani.

**Writing – review & editing:** Estu Meilani, Asfarina Zanudin, Nor Azlin Mohd Nordin.
